# 血清和血清外泌体的蛋白质组分析及其在肝内胆管癌中的应用

**DOI:** 10.3724/SP.J.1123.2021.04009

**Published:** 2021-11-08

**Authors:** Kaige YANG, Weiwei WANG, Yan WANG, Chao YAN

**Affiliations:** 上海交通大学药学院, 上海 200240; School of Pharmacy, Shanghai Jiao Tong University, Shanghai 200240, China; 上海交通大学药学院, 上海 200240; School of Pharmacy, Shanghai Jiao Tong University, Shanghai 200240, China; 上海交通大学药学院, 上海 200240; School of Pharmacy, Shanghai Jiao Tong University, Shanghai 200240, China; 上海交通大学药学院, 上海 200240; School of Pharmacy, Shanghai Jiao Tong University, Shanghai 200240, China

**Keywords:** 液相色谱-质谱, 肝内胆管癌, 蛋白质组学, 统计模型, 差异蛋白质, 外泌体, 血清, liquid chromatography-mass spectrometry (LC-MS), intrahepatic cholangiocarcinoma, proteomics, statistical model, differential protein, exosome, serum

## Abstract

外泌体是由各种类型细胞在正常或非正常生理情况下分泌释放至细胞外且携带多种生物活性分子的细胞外囊泡,在细胞间通讯和免疫应答等生物过程中发挥着重要作用。肝内胆管癌是一种胆道上皮恶性肿瘤,早期无明显临床症状且生存率较低,目前常用的诊断手段包括依赖于影像设备的诊断方式和灵敏度及特异性较低的诊断标志物等,这些手段的不足对发展新的特异性标志物提出了需求。该文对血清中的外泌体进行了分离和表征,并采用液相色谱-质谱技术针对健康组与肝内胆管癌患者组的血清样本和血清外泌体样本进行了无标记定量蛋白质组学分析,分别从两种类型样本中鉴定并筛选到271和430种可信蛋白质。基于血清样本和血清外泌体样本的可信蛋白质定量表达值进行多维统计分析都能将健康组与肝内胆管癌患者组良好地区分开。对血清样本中鉴定到的蛋白质进行差异蛋白质筛选,肝内胆管癌患者组相对于健康组有15个上调和8个下调蛋白质;对血清外泌体样本中鉴定到的蛋白质进行差异蛋白质筛选,肝内胆管癌患者组相对于健康组有33个上调和18个下调蛋白质;基于两种样本筛选到的差异蛋白质中仅有4个是重复的,且基于血清外泌体样本的51个差异蛋白质中有35个蛋白质属于外泌体蛋白质数据库。针对差异蛋白质进行生物学信息分析,与差异蛋白质相关的分子功能、生物过程和信号通路主要涉及天然免疫反应、炎症反应和凝血等过程。该研究为发现肝内胆管癌的潜在生物标志物和探究肝内胆管癌的发生、发展和转移等过程提供了参考和借鉴价值。此外,通过比较研究发现血清外泌体样本能够获得较多的差异蛋白质和生物学信息,证明了外泌体作为组学分析样本的价值和应用潜力。

## Abstract (en)

Exosomes are extracellular vesicles with a diameter in the range of 50-200 nm and a double-layer lipid membrane structure that are released by various types of cells under normal or abnormal physiological conditions. At present, according to their extensive biological functions, exosomes have been used in a wide range of research fields and applications, including as potential biomarkers and drug delivery vehicles. Intrahepatic cholangiocarcinoma is a malignant tumor of the biliary epithelium with the characteristics of cholangiocellular differentiation, which accounts for 10%-15% of all types of primary liver cancer. Intrahepatic cholangiocarcinoma has no obvious clinical symptoms in the early stages, which results in a low survival rate. Imaging equipment dependent diagnostic methods and currently commonly used diagnostic markers with low sensitivity/specificity have necessitated the development of new specific markers for intrahepatic cholangiocarcinoma.

In this study, exosomes were isolated from serum using a commercial kit and characterized through nanoparticle tracking analysis, Western blotting analysis, and transmission electron microscopic analysis to prove the successful isolation of exosomes. Sodium dodecyl sulfate-polyacrylamide gel electrophoresis (SDS-PAGE) showed that the protein profiles of the serum and serum exosome samples were significantly different. In particular, some high-abundance proteins in the serum samples were significantly reduced or disappeared in the serum exosome sample. Meanwhile, some protein bands (which may belong to exosomes) that did not appear in the serum samples appeared in the serum exosome samples, leading to the conduciveness of subsequent mass spectrometry analysis. The serum and serum exosome samples of the healthy control and intrahepatic cholangiocarcinoma groups were analyzed by liquid chromatography-mass spectrometry for label-free quantitative proteomics. In total, 547 proteins were identified in the serum exosome samples, of which 341 (more than 60%) could be found in the exosomal protein database. In addition, 271 and 430 credible proteins were screened from the serum and serum exosome samples for multi-dimensional statistical analysis and differential protein discovery. Unsupervised principal component analysis and supervised orthogonal partial least squares discriminant analysis based on the quantitative proteome of the serum and serum exosome samples could distinguish the healthy control and intrahepatic cholangiocarcinoma groups well, which illustrates that the two types of samples both have potential in the diagnosis of intrahepatic cholangiocarcinoma. There were 15 upregulated and 8 downregulated proteins screened in the intrahepatic cholangiocarcinoma group compared to the healthy control group based on the serum samples, while 33 upregulated and 18 downregulated proteins were screened in the intrahepatic cholangiocarcinoma group compared to the healthy control group based on the serum exosome samples, and only four of the differential proteins screened based on the two types of samples were duplicates. At the same time, 35 of the 51 differentially expressed proteins screened based on serum exosome samples belonged to the exosomal protein database. Finally, biological information analysis was performed according to these differential proteins. The molecular functions, biological processes, and signal pathways enriched by these differential proteins mainly involved the innate immune responses, inflammatory responses, and blood coagulation. This study provides a reference value for potential biomarker discovery and exploration of the process of occurrence, development, and metastasis of intrahepatic cholangiocarcinoma. Moreover, compared with proteomic analysis based on serum samples, proteomic analysis based on serum exosome samples can be used to identify more differential proteins and biological information, and although these differential proteins and biological information may show big differences, the specificity and sensitivity of exosome-based diagnosis and the superiority of exosomes as samples for proteomic analysis has proven the application value of exosomes.

外泌体(exosomes)是一种直径范围在50~200 nm且具有双层脂质膜结构的细胞外囊泡。由各种类型细胞在正常或非正常生理情况下释放至细胞外,并存在于血液、尿液、组织液和脑脊液等体液中^[1,2,3]^。外泌体携带多种生物活性物质,如核酸、蛋白质和小分子代谢物等,并且能够通过外泌体把这些生物活性物质由母细胞转移至靶细胞,以此在各种正常和非正常生理状态下传递细胞间信息,即通过传递遗传物质、转移受体、信号分子等方式影响其他细胞^[4,5]^。因此,外泌体在细胞间通讯^[6,7]^、免疫应答^[8,9,10]^、代谢途径^[11,12,13]^和肿瘤发生、发展及转移^[7,8,14]^等过程中都发挥着重要的作用。目前,基于外泌体广泛的生物学作用,将外泌体用于疾病的诊断和治疗的研究与应用越来越多^[15]^,如发现潜在生物标志物^[16,17,18]^和用作药物传递载体^[17,19]^等。

胆管癌(cholangiocarcinoma, CCA)是一种胆道上皮恶性肿瘤,表现出胆管细胞分化的特征,占所有原发性肝癌类型的10%~15%,是第二大常见的原发性肝癌。CCA在解剖学上可分为肝内胆管癌(intrahepatic cholangiocarcinoma, iCCA)、肝门周围胆管癌(perihilar cholangiocarcinoma, pCCA)和远端胆管癌(distal cholangiocarcinoma, dCCA)^[20]^。由于大多数CCA患者早期没有明显症状,被发现时已处于晚期,治愈性手术仅在10%~15%的病例中可行,且手术切除后的复发率超过60%。因此,CCA总体疾病预后较差,所有患者的5年生存率仅为5%~15%^[21]^。当前CCA的诊断主要依靠影像技术,包括超声、计算机断层扫描(CT)、磁共振成像(MRI)以及先进的内镜胆道成像技术^[22]^。但是CCA肿瘤类似物(脓肿、血管瘤和融合性肝纤维化等)会对影像诊断造成较大的影响^[23]^。CCA最常用的血清诊断标志物是碳水化合物抗原19-9(CA19-9)和癌胚抗原(CEA),同时也是术后监测的有力工具。但是,CA 19-9和CEA的灵敏度及特异性都较低^[22,24,25]^。因此,急需发展新的特异性生物标志物用于CCA的筛查和早期诊断,促进CCA的治疗。

血清和外泌体中都含有丰富的蛋白质,是良好的基于蛋白质组学的潜在生物标志物发现样本之一^[26]^。因此,本文选择正常志愿者和CCA亚型中的iCCA患者的血清样本,从中提取外泌体并进行表征验证;然后基于液相色谱-质谱(LC-MS)的无标记定量蛋白质组学技术,对两组样本的血清和血清来源外泌体分别进行分析和统计处理,筛选出疾病状态下差异表达的蛋白质,从蛋白质水平理解iCCA肿瘤发生、发展及转移等过程,为iCCA的早期诊断和潜在生物标志物发现提供参考和借鉴。此外,比较血清和血清来源外泌体这两类样本获得的蛋白质组信息,探究不同类型的样本在组学分析中的差异。

## 1 实验部分

### 1.1 血清样本的收集

本研究中所用健康志愿者对照组(HC)及iCCA患者血清样本均由第二军医大学附属东方肝胆医院提供。所有采集的HC和iCCA患者临床样本均与提供者签署了书面知情同意书,并经伦理委员会批准。本实验共采集了6例HC血清样本和6例iCCA患者血清样本。采集的外周血样本在室温下静置1 h,然后以2000g离心10 min,取上清液血清置于-80 ℃下保存直至使用。

### 1.2 仪器、试剂与材料

nLC1200-Q Exactive Plus纳升液相色谱-四极杆轨道阱质谱仪(Thermo Fisher Scientific,美国);ZetaView纳米颗粒跟踪分析仪(NTA, Particle Metrix,德国);JEM-2100F场发射透射电子显微镜(TEM, JEOL,日本); Centrifuge 5417R冷冻离心机(Eppendorf,德国);电泳仪、电泳槽、转膜槽、Tanon 1600全自动凝胶图像分析系统和Tanon 4600全自动化学发光/荧光图像分析系统(Tanon,中国); FiveEasy^TM^ pH计(Mettler Toledo,瑞士)。

Umibio外泌体提取纯化试剂盒(Cat. No: UR52136, Umibio,中国); Radio Immunoprecipitation Assay (RIPA)裂解液、100 mmol/L苯甲基磺酰氟(PMSF)蛋白酶抑制剂、考马斯亮蓝G250、三羟甲基氨基甲烷(Tris)、甘氨酸、十二烷基硫酸钠(SDS)、SDS-聚丙烯酰胺凝胶电泳(PAGE)蛋白上样缓冲液(5×)、30%Acr-Bis(29:1)制胶液、1.5 mmol/L Tris-HCl缓冲液(pH 8.8)、1 mmol/L Tris-HCl缓冲液(pH 6.8)、四甲基乙二胺(TEMED)、BeyoColor^TM^彩色预染蛋白相对分子质量标准(10~170 kD)、吐温-20(Tween-20)、CD9 兔单克隆抗体、CD63兔单克隆抗体、CD81兔单克隆抗体、辣根过氧化物酶标记山羊抗兔IgG(H+L)、化学发光试剂盒、BCA蛋白质浓度测定试剂盒均购自上海碧云天生物技术有限公司;牛血清白蛋白(bovine serum albumin, BSA)购自北京伊诺凯科技有限公司;磷钨酸负染色液(2%)购自上海泰坦科技股份有限公司;分析纯丙酮、甲醇、乙醇、甲酸、盐酸、氯化钠、氯化钾、磷酸氢二钠、磷酸二氢钾购自国药集团化学试剂有限公司;质谱级乙腈、甲酸购自Thermofisher Scientific(美国); LC-MS级碳酸氢铵、二硫苏糖醇(≥98%)、碘乙酰胺(98%)购自Sigma-Aldrich(美国);测序级修饰胰蛋白酶购自Promega Corporation(美国)。

聚偏二氟乙烯(PVDF)膜(Merck,Germany); 0.22 μm亲水针式滤器(Anpel,中国); 10 kDa超滤管(Sartorius,德国); C18脱盐柱(GL Science,日本)。

### 1.3 血清外泌体的分离

血清预处理:将-80 ℃冻存的血清样本解冻后通过0.22 μm滤膜过滤。过滤后的血清样本于4 ℃以3000g离心10 min,去除样本中的细胞碎片;将上清液转移至新的离心管中,于4 ℃以10000g离心10 min,进一步去除样本中的杂质和较大的细胞外囊泡,取上清液。然后将经过预处理的血清样本分为两份,一份用于外泌体提取,一份用于血清蛋白质组样本提取。

外泌体分离:将一份经过预处理的血清样本按照血清样本、磷酸盐缓冲液(PBS,配制方法:8 g氯化钠、0.2 g氯化钾、1.44 g磷酸氢二钠、0.24 g磷酸二氢钾、1 L纯水,盐酸调pH至7.4)、Blood PureExo Solution(体积比为1:3:1)混合,涡旋振荡混匀1 min后置于4 ℃静置2 h。静置后将上述混合液于4 ℃以10000g离心60 min,弃去上清液,并取适量PBS重悬沉淀,并将重悬液于4 ℃以12000g离心2 min,取上清液,重复2~3次,获得的上清液为外泌体粗制剂。将收获的外泌体粗制剂转移入Exosome Purification Filter柱上室中,于4 ℃以3000g离心10 min,离心后收集柱管底液体,即为纯化后的外泌体溶液,将其置于-80 ℃下保存直至使用。

取部分分离得到的血清外泌体,加入相同体积的RIPA裂解液和1%(体积分数)的100 mmol/L PMSF蛋白酶抑制剂,混匀后置于冰上裂解60 min,于4 ℃以10000g离心5 min后取上清液,即为外泌体蛋白质溶液。

### 1.4 外泌体的表征

1.4.1 纳米粒径分析

通过NTA分析外泌体的粒径分布范围。首先在样品池中注入标准物质(粒径为100 nm)校准仪器;用PBS冲洗样品池后,将经PBS适当稀释的外泌体样品注入样品池以测量粒径,结果以每mL溶液颗粒数累积值为纵坐标,粒径值(单位为nm)为横坐标,即particles/mL (sum)-diameter (nm)的形式显示。

1.4.2 蛋白质免疫印迹分析

采用蛋白质印迹法对血清外泌体的标志性蛋白质进行检测,并用仅经预处理的血清样品作为对照。简要步骤如下:HC组和iCCA组的血清及血清外泌体蛋白质溶液通过BCA试剂盒测定蛋白质浓度,分别各取10 μg蛋白质与上样缓冲液混合并在100 ℃煮沸后,上样到10% SDS-PAGE凝胶中,先在90 V电压下电泳30 min,然后在120 V电压下电泳约60 min。将凝胶上的蛋白质转移到预先用甲醇活化好的PVDF膜上(湿转法,恒流200 mA, 90 min)。转膜结束后取出PVDF膜,用TBST(Tris-HCl-Tween缓冲盐溶液,配制方法:3 g Tris, 8.8 g氯化钠,1 L纯水,1 mL Tween-20)配制的5%(质量分数)BSA在室温、50 r/min下封闭1 h,封闭后将PVDF膜分别与CD9、CD63和CD81抗体在4 ℃下共孵育过夜。将一抗孵育过夜后的PVDF膜用TBST溶液洗涤3次,加入辣根过氧化物酶偶联的二抗,在室温、50 r/min下孵育2 h。二抗孵育后的PVDF膜用TBST洗涤3次后,加入显影液并通过自动化学发光图像分析系统拍摄图像。

1.4.3 透射电镜分析

将10 μL血清外泌体溶液滴加在200目高聚碳膜铜网上,并静置10 min以充分吸附。用滤纸吸去多余的液体,滴加10 μL 2%磷钨酸进行2~3 min的负染色。用滤纸吸去多余的液体,在室温下干燥后通过TEM进行形貌观察。

### 1.5 基于考马斯亮蓝染色的聚丙烯酰胺凝胶电泳

分别取HC和iCCA的血清样品和血清外泌体蛋白质溶液样品中10 μg蛋白质,并分别与上样缓冲液混合后在100 ℃煮沸,上样到10% SDS-PAGE凝胶中,先在90 V电压下电泳30 min,然后在120 V电压下电泳约60 min。电泳结束后,将SDS-PAGE凝胶置于考马斯亮蓝染色液中染色并脱色后,拍摄图像。

### 1.6 蛋白质组样品处理

分别取每个HC和iCCA的血清样品和血清外泌体蛋白质溶液样品中50 μg蛋白质,加入6倍体积的蛋白沉淀剂(乙醇-丙酮-甲酸,50:50:0.1,v/v/v),在-20 ℃下过夜。过夜后溶液于4 ℃以10000g离心60 min,弃去上清液,沉淀用90%(v/v)丙酮水溶液清洗2遍,于室温晾干10 min,将沉淀溶解于200 μL 100 mmol/L碳酸氢铵水溶液中。然后在蛋白质溶液中加入2 μL 1 mol/L二硫苏糖醇溶液,于37 ℃孵育还原1 h;加入10 μL 1 mol/L碘乙酰胺溶液,于室温避光烷基化40 min。将上述液体转移至10 kDa超滤管中,于4 ℃以10000g离心20 min,弃去滤液,加入50 mmol/L碳酸氢铵,于4 ℃以10000g离心20 min,弃去滤液,重复3次,然后按酶和蛋白1:25(质量比)加入胰蛋白酶,于37 ℃酶解过夜。酶解后,于4 ℃以10000g离心20 min,收集滤液。上述滤液经C18脱盐柱脱盐后干燥,用10 μL 0.1%甲酸水溶液复溶多肽后进行质谱分析。

### 1.7 基于LC-MS的蛋白质组分析

多肽样品通过配备有电喷雾电离(ESI)纳米喷雾源的nLC1200-Q Exactive Plus仪的自动进样器进入C18柱中(50 cm×75 μm, 3 μm)。流动相A相是0.1%甲酸水溶液;流动相B相是0.1%甲酸-80%乙腈-20%水溶液。总流速是300 nL/min,梯度如下:0~2 min, 2%B~6%B; 2~95 min, 6%B~20%B; 95~107 min, 20%B~32%B; 107~108 min, 32%B~100%B; 108~110 min, 100%B。扫描模式是正模式,离子源喷雾电压设置为1.8 kV,毛细管温度设置为275 ℃。质谱一级扫描质量数范围为*m/z* 350~1800,分辨率为70000;自动增益目标值(AGC)为3×10^6^,最大离子注入时间(MIT)为50 ms。二级扫描(dd-MS^2^/dd-SIM)的分辨率为17500, AGC为1×10^5^, MIT为45 ms。选取丰度前20的肽段进行二级裂解,碎裂模式为高能碰撞解离(HCD),碰撞能(NCE)设置为28,离子排除是排除电荷为1、7、8和大于8的离子,动态排除时间设为30 s。使用软件Proteome Discoverer 2.5(ThermoFisher Scientific)对原始LC-MS/MS数据文件进行分析,并根据Uniprot人类数据库搜索谱图(sequence 20148, https://www.uniprot.org/)。数据库搜索参数设置为固定修饰为氨基甲酰甲基(C),可变修饰为氧化(M)、脱酰胺(N, Q)和乙酰化(N-末端);初始前体离子和碎片离子的质量容差分别为1×10^-5^ Da和0.02 Da。最多允许2个胰蛋白酶漏切位点。肽鉴定的过滤设置为至少检测到2个特征肽段且假阳率(FDR)小于1%。

### 1.8 蛋白质组数据分析

对HC和iCCA的血清样品中鉴定到的蛋白质以及HC和iCCA的血清外泌体样品中鉴定到的蛋白质分别进行相对定量比较。对于数据库检索得到的原始数据,保留HC和iCCA组中表达率均≥50%的蛋白质的表达值(或一组表达率为0,另一组表达率≥50%),并用同组样品的平均值填充缺失值,获得可信蛋白表达值。对可信蛋白表达值进行中位数归一化和log_2_对数转换,获得可信蛋白质表达值标准化数据,并用可信蛋白表达值标准化前后数据(可信蛋白标准化前的表达值以log_2_ intensity形式展示)绘制箱线图。并根据可信蛋白质表达值标准化数据进行多维统计分析,通过无监督的主成分分析(PCA)和有监督的正交偏最小二乘判别分析(OPLS-DA)观察基于血清或血清外泌体的无标记定量蛋白质组能否区分HC和iCCA。

对可信蛋白表达值的中位数归一化数据使用GraphPad Prism 8软件通过moderate t-statistic计算*P*值,并基于*P*值<0.05和差异倍数(fold change, FC)值<0.5 (下调)或>2 (上调)筛选出HC和iCCA组之间差异表达的蛋白质。通过韦恩图比较鉴定到的血清外泌体蛋白质和ExoCarta外泌体蛋白质库(http://www.exocarta.org);比较基于血清外泌体样品筛选得到的iCCA组相对HC组的差异蛋白质和ExoCarta外泌体蛋白质库;比较基于血清样品和基于血清外泌体样品筛选得到的iCCA组相对HC组的差异蛋白质。最后,基于免费的在线数据库分析差异表达蛋白质的生物学信息,包括基因本体论(GO)富集分析(根据-log_10_ FDR排序,展示每个类别的top10, https://string-db.org/)和京都基因与基因组百科全书(KEGG)通路分析(根据-log_10_ *P*-value排序,展示top15; http://kobas.cbi.pku.edu.cn/kobas3/genelist/),并根据获得的生物学信息,使用GraphPad Prism 8和Origin 2019b软件绘制条形图和气泡图。

## 2 结果与讨论

### 2.1 血清外泌体的表征

为了验证通过外泌体提取纯化试剂盒能够从血清中成功提取获得外泌体,对分离提取的血清外泌体分别进行纳米粒径分布分析、标志性蛋白免疫印迹分析和透射电镜分析,结果见图1。从图1a和1b的外泌体粒径分布结果可知,HC血清中提取的外泌体粒径的*X*_10_、*X*_50_和*X*_90_(颗粒从小到大排列,分别包含前10%、50%和90%颗粒数时的粒径值)分别为78.4、141.9和221.0 nm,峰值在164.9 nm; iCCA血清中提取的外泌体粒径的*X*_10_、*X*_50_和*X*_90_分别为80.8、146.4和238.4 nm,峰值在171.0 nm,基本都符合定义中外泌体的粒径分布范围^[1,2]^。

**图1:**
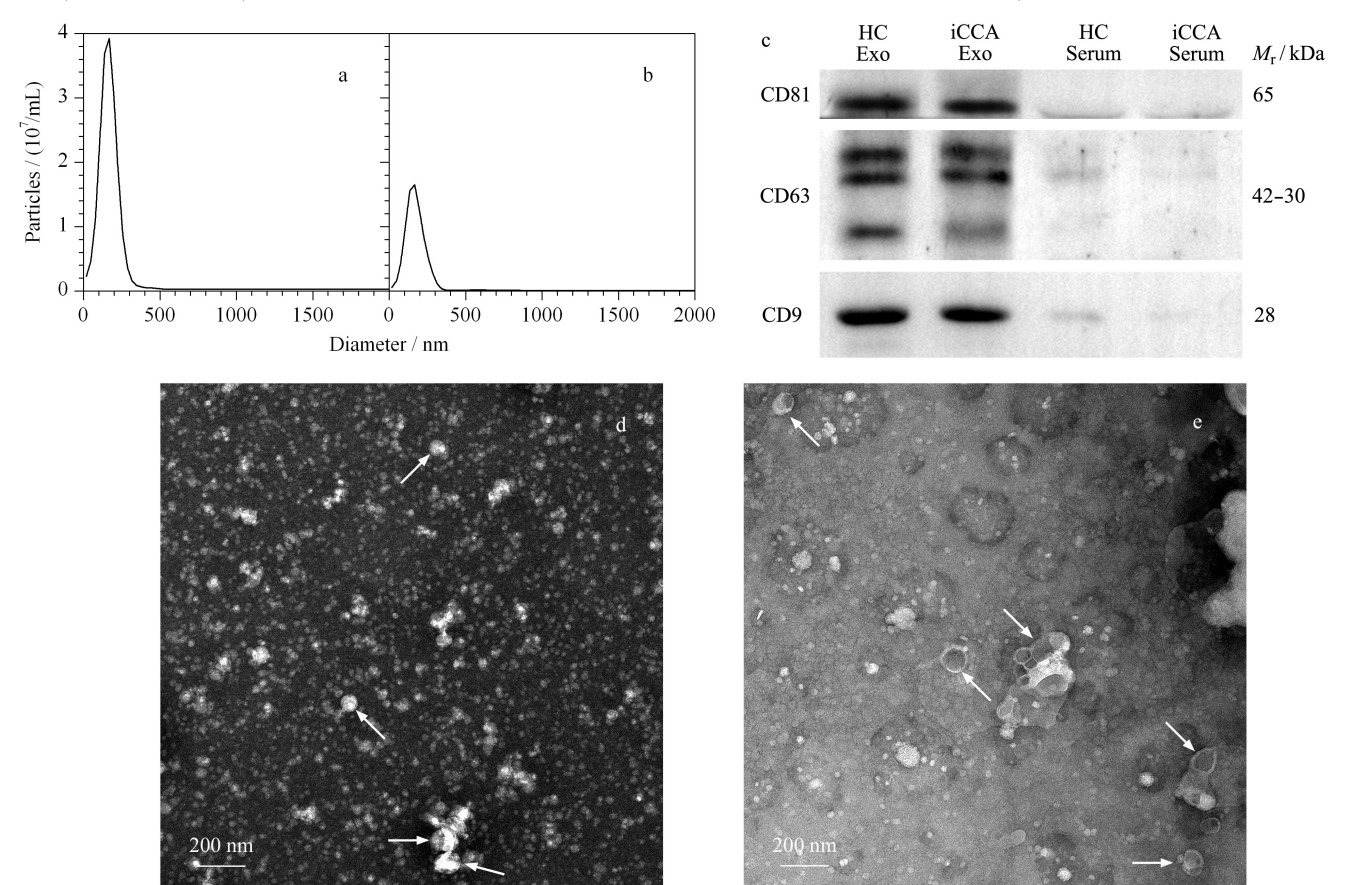
血清外泌体的表征

然后,采用蛋白质印迹法验证分离提取的血清外泌体样品中外泌体标志性蛋白CD9、CD63和CD81的存在,同时以仅经预处理的血清样品作为对照,如图1c所示,在从HC和iCCA血清提取的外泌体样品中(图1c中HC Exo和iCCA Exo泳道),都能够检测到标志性蛋白CD9、CD63和CD81的存在;而对于HC和iCCA的血清样品(图1c中HC Serum和iCCA Serum泳道),仅能观察到微弱的条带甚至没有条带,表明尽管血清中存在外泌体,但是由于外泌体的浓度较低,因此无法通过蛋白质印迹法检测到外泌体标志性蛋白。同时,外泌体样品中标志性蛋白质的高含量也进一步证明了血清中的外泌体获得了富集和分离。

最后,对HC和iCCA血清中提取的外泌体样品进行了TEM分析,如图1d和1e所示,能够观察到直径在100 nm左右且具有经典的茶托样结构的囊泡(图1d和1e中箭头所指),符合外泌体的形貌特征^[1,27]^。但是,同样能够观察到许多不具备外泌体经典结构的圆形物质(直径范围在50~200 nm左右),这些物质可能是由于过度负染色而无法在透射电镜下呈现出经典结构的外泌体,也可能是一些与外泌体共分离的杂质,如与外泌体粒径范围有重叠的极低密度脂蛋白(VLDL, 30~80 nm, 0.930~1.006 g/cm^3^)和乳糜微粒(CM, 75~1200 nm, <0.930 g/cm^3^)等^[28]^。我们知道,获取纯净的外泌体目前还非常困难^[29]^,所以尽管该外泌体分离方法会引入一些杂质,但是纳米粒径分布分析、标志性蛋白免疫印迹分析和透射电镜分析的结果都表明血清外泌体得到了较好的富集分离,所获得的血清外泌体可以用于后续的蛋白质组学分析^[30]^。

### 2.2 血清外泌体的分离效果评价

首先采用SDS-PAGE对提取的外泌体中的蛋白质进行初步的分析,同时以血清蛋白质作为对照,结果如图2a所示。从SDS-PAGE图像中可以观察到,血清外泌体的蛋白质轮廓与血清蛋白质有较大的区别,特别是一些血清中高丰度的蛋白质在外泌体中有明显的减少或者消失,如66 kDa的白蛋白(albumin)。但是,血清和血清外泌体泳道上的条带也有一些相似点,即一些杂质如免疫球蛋白未能有效除去,因此在外泌体中也可以观察到免疫球蛋白IgG的重链(约50 kDa)和轻链(约25 kDa)的条带(可能是由于免疫球蛋白与外泌体膜表面的蛋白质等分子发生相互作用而导致的共分离^[31]^)。此外,外泌体中出现了一些血清中没有的蛋白质条带,这些可能来源于外泌体内含的蛋白质相对含量的提高,这有利于后续的质谱分析。

**图2:**
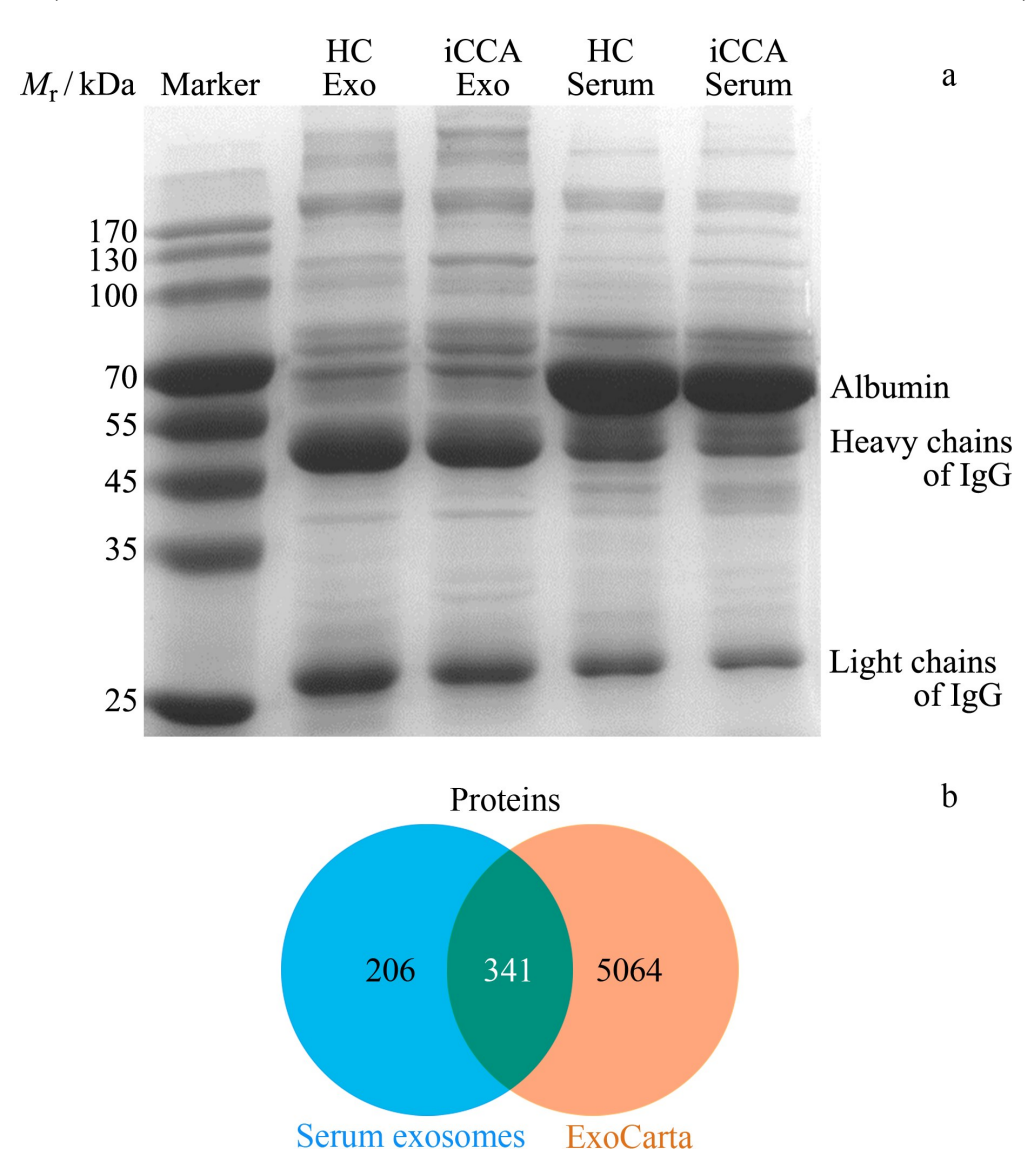
血清外泌体的蛋白质组分析

进一步利用LC-MS对外泌体中的蛋白质组进行分析,在12个外泌体样品中共鉴定到了547种蛋白质,其中有341种蛋白质归属于外泌体蛋白质数据库ExoCarta,如图2b所示。超过60%的鉴定蛋白质归属于外泌体蛋白质数据库也进一步证明了外泌体的成功富集以及在蛋白质组学中的应用价值。

### 2.3 血清蛋白质组和血清外泌体蛋白质组在肝内胆管癌中的应用

通过LC-MS对HC与iCCA组的血清和血清外泌体的蛋白质组分别进行无标记定量分析,并将其应用于iCCA的诊断、潜在标志物筛选和生物信息分析,同时比较血清和血清外泌体两种类型的样本在基于蛋白质组的iCCA研究中的差异。

血清和血清外泌体中分别鉴定到317和547种蛋白质,经过筛选后分别获得271和430种可信蛋白质。对可信蛋白质的定量表达值数据进行标准化处理,并通过箱线图展示标准化前后的数据,如图3所示。标准化数据(图3b)较标准化前数据(图3a)趋于中心位置,表明标准化处理可减少强度分布之间的差异并确保样品之间的可比性,使得标准化数据有着更好的质量,更加有利于统计学分析。

**图3:**
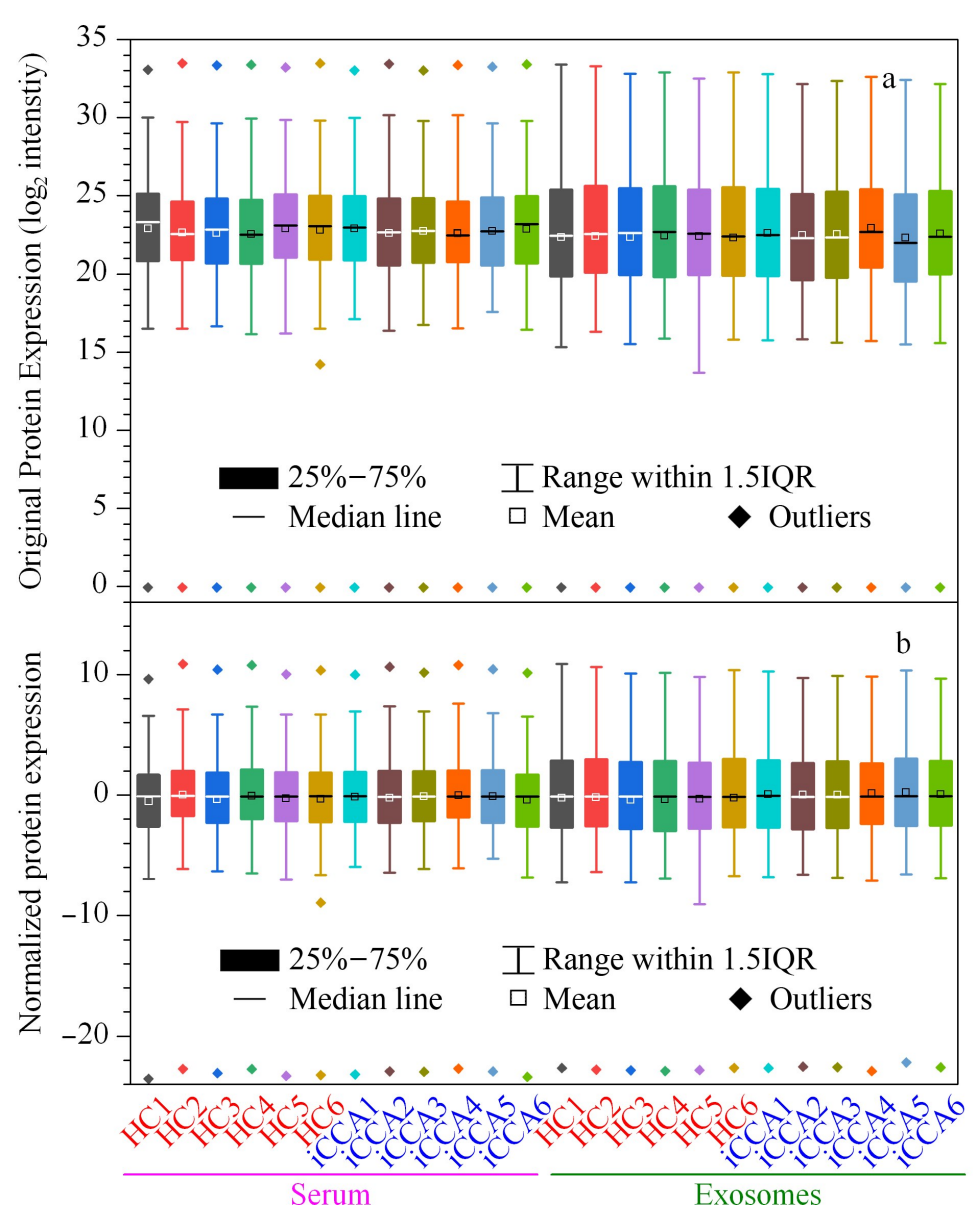
可信蛋白质定量表达值数据标准化处理(a)前、 (b)后的箱线图

利用标准化蛋白质定量表达值数据对HC与iCCA组进行多维统计分析,血清和血清外泌体样本的PCA得分图(见图4a、4b)和OPLS-DA得分图(见图4c、4d)都显示出HC与iCCA组能够得到良好的区分。同时,由OPLS-DA模型参数(见表1)可知,*R*^2^*Y*(cum)和*Q*^2^(cum)均大于0.9,表明两种类型样本的OPLS-DA模型拟合的良好性。也说明本方法提取的血清外泌体的定量蛋白质组能够较好地区分HC与iCCA组以及预测未知样本的归属,在iCCA诊断应用中拥有潜在优势。

**图4:**
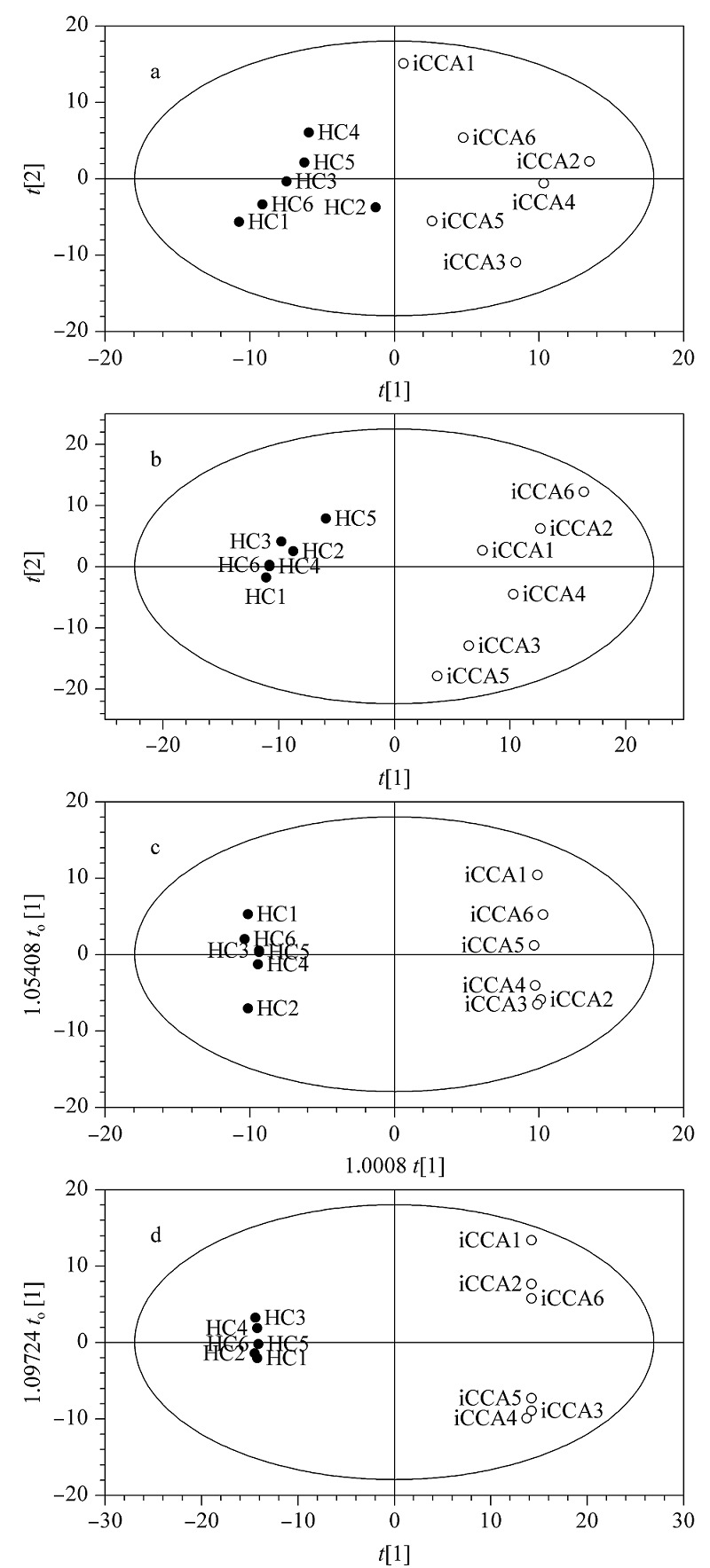
基于标准化定量蛋白质组的多维统计分析

**表1:** 基于血清和血清外泌体标准化定量蛋白质组的 OPLS-DA模型参数

Sample type	A	R^2^X(cum)	R^2^Y(cum)	Q^2^(cum)
Serum	1P+1O	0.509	0.999	0.969
Exosome	1P+1O	0.547	0.999	0.984

*A*: number of components; P: predictive component; O: orthogonal component; *R*^2^*X*(cum) and *R*^2^*Y*(cum): cumulative modeled variations in the *X* and *Y* matrix; *Q*^2^(cum): cumulative predicted variation in the *Y* matrix.

分别从血清和血清外泌体两组样本中筛选HC与iCCA组的差异表达蛋白质。根据FC值和*P*值两个参数进行差异蛋白质筛选,在血清样本中(见图5a), iCCA组相对于HC组有15个上调和8个下调的蛋白质(见表2);在血清外泌体样本中(见图5b), iCCA组相对于HC组有33个上调和18个下调的蛋白质(见表2),并且在基于血清外泌体筛选到的51个差异蛋白质中,有35个蛋白质归属于外泌体蛋白质数据库(见图5c)。同时,基于血清外泌体筛选到的51个差异蛋白质中,IGHA2、IGHG4和PIGR等蛋白质(都归属于外泌体蛋白质数据库)也已被相关文献^[32]^报道在健康志愿者和CCA患者的细胞外囊泡(基于超速离心法从血清中分离)中差异表达,受试者工作特征曲线(ROC曲线)的曲线下面积(AUC)分别为0.824、0.818和0.844,作为生物标志物能够表现出良好的诊断能力。

**表2:** 基于血清和血清外泌体样本的HC与iCCA组的差异蛋白质

Serum					Exosome			
Accession	Gene symbol	FC(iCCA/HC)	P-value		Accession	Gene symbol	FC(iCCA/HC)	P-value
P00450	CP	2.2429	0.0053		P04275	VWF	2.1460	0.0383
P06681	C2	2.2294	0.0001		P01009	SERPINA1	2.4817	0.0001
P01833	PIGR	12.4548	0.0091		P01877	IGHA2	+∞	0.0019
Q06033	ITIH3	2.5237	0.0086		P01833	PIGR	9.5927	0.0048
P36980	CFHR2	3.9305	0.0371		P20742	PZP	3.7771	0.0034
P00742	F10	2.2807	0.0001		P36980	CFHR2	2.1296	0.0109
Q04756	HGFAC	2.5523	0.0024		P01011	SERPINA3	2.3901	0.0002
P05362	ICAM1	+∞	0.0004		P02763	ORM1	2.3329	0.0028
Q9BYE9	CDHR2	+∞	0.0005		P15144	ANPEP	2.0323	0.0167
A0A0A0MRZ9	IGLV5-52	2.1752	0.0147		P68133	ACTA1	2.1930	0.0357
P19320	VCAM1	3.1375	0.0206		P02775	PPBP	2.0735	0.0422
P13473	LAMP2	2.1709	0.0220		Q04695	KRT17	+∞	0.0016
Q9BT22	ALG1	2.0182	0.0281		Q14766	LTBP1	2.0226	0.0274
Q8NFU5	IPMK	12.1589	0.0006		Q6UWP8	SBSN	4.0805	0.0177
A2VCL2	CCDC162	2.4897	0.0018		P07327	ADH1A	+∞	0.0052
P06727	APOA4	0.4316	0.0030		P22792	CPN2	2.8882	0.0049
P07996	THBS1	0.4055	0.0021		P35555	FBN1	2.4629	0.0282
A0A075B6S6	IGKV2D-30	0.0000	0.0288		P02750	LRG1	2.4234	0.0016
P01602	IGKV1-5	0.0000	0.0006		P20930	FLG	+∞	0.0165
P13647	KRT5	0.3018	0.0392		P08571	CD14	3.7509	0.0234
Q7Z794	KRT77	0.3075	0.0094		Q9UEW3	MARCO	2.4943	0.0434
P67936	TPM4	0.0000	0.0110		P02792	FTL	+∞	0.0000
O60229	KALRN	0.4881	0.0039		P21333	FLNA	2.0853	0.0431
					P28066	PSMA5	+∞	0.0000
					P12814	ACTN1	3.5309	0.0364
					Q9UGM3	DMBT1	2.4154	0.0404
					Q9H4G4	GLIPR2	3.8794	0.0397
					P78509	RELN	2.6213	0.0149
					Q15063	POSTN	+∞	0.0000
					P30101	PDIA3	+∞	0.0070
					O14818	PSMA7	2.2647	0.0024
					Q7Z398	ZNF550	2.8163	0.0197
					O95810	CAVIN2	+∞	0.0001
					P01861	IGHG4	0.3768	0.0009
					P06396	GSN	0.4789	0.0032
					P06727	APOA4	0.2324	0.0000
					Q16610	ECM1	0.2677	0.0001
					P04070	PROC	0.2334	0.0437
					P35443	THBS4	0.3062	0.0060
					A0A075B6R2	IGHV4-4	0.3530	0.0112
					P22105	TNXB	0.3142	0.0009
					A0A0B4J2H0	IGHV1-69D	0.1628	0.0016
					Q04756	HGFAC	0.3811	0.0005
					Q14532	KRT32	0.0000	0.0007
					A0A0G2JMI3	IGHV1-69-2	0.3718	0.0104
					A0A087WSZ0	IGKV1D-8	0.0000	0.0002
					P12109	COL6A1	0.4603	0.0094
					Q68EA5	ZNF57	0.4689	0.0425
					Q96MV8	ZDHHC15	0.3387	0.0171
					Q13939	CCIN	0.3101	0.0093
					Q9BS31	ZNF649	0.0000	0.0021

**图5:**
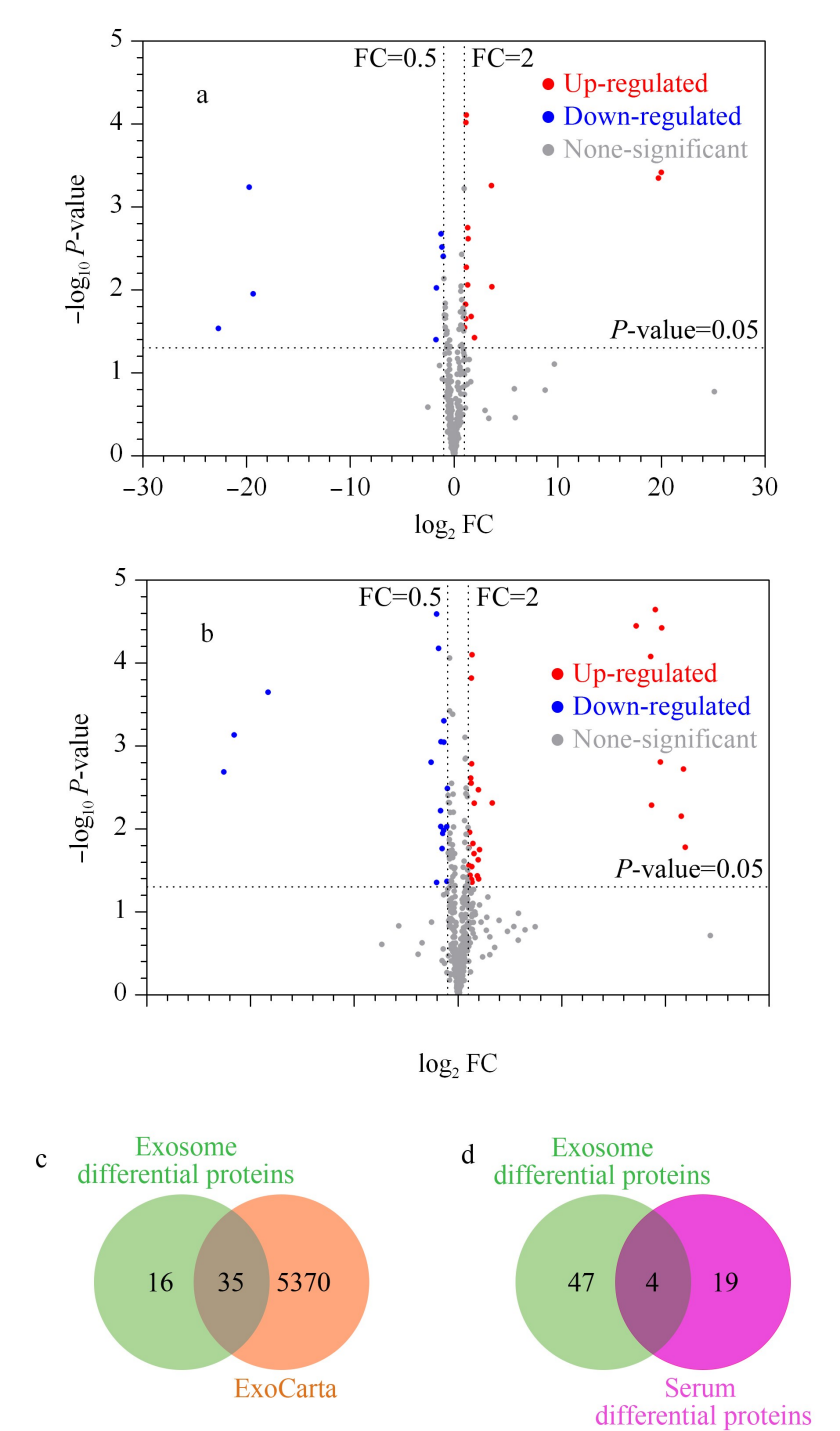
基于血清和血清外泌体样本的HC与iCCA组的差异蛋白质筛选

此外,基于血清筛选到的23个差异蛋白质和基于血清外泌体筛选到的51个差异蛋白质中,仅有4个蛋白质是重复的(见图5d),表明了每种样本在潜在标志物发现过程中的独特性。同时,基于血清外泌体筛选获得的差异蛋白质数目多于血清,说明外泌体作为一种新的潜在生物标志物发现的样本模式,有可能获得更多的生物学信息,对解释疾病机制更具备参考价值。

最后,对筛选获得的差异蛋白质进行生物信息分析,包括GO分析和KEGG通路分析,如图6所示。这些蛋白质富集到的分子功能、生物过程和信号通路主要涉及天然免疫反应、炎症反应和凝血等,如基于两种类型样本差异蛋白质富集到的共同生物过程——血小板脱粒(platelet degranulation);基于血清样本差异蛋白质独特富集到的NF-κB信号通路(NF-kappa B signaling pathway);以及基于血清外泌体样本差异蛋白质独特富集到的中性粒细胞脱粒(neutrophil degranulation)生物过程及补体和凝血级联(complement and coagulation cascades)信号通路。

**图6:**
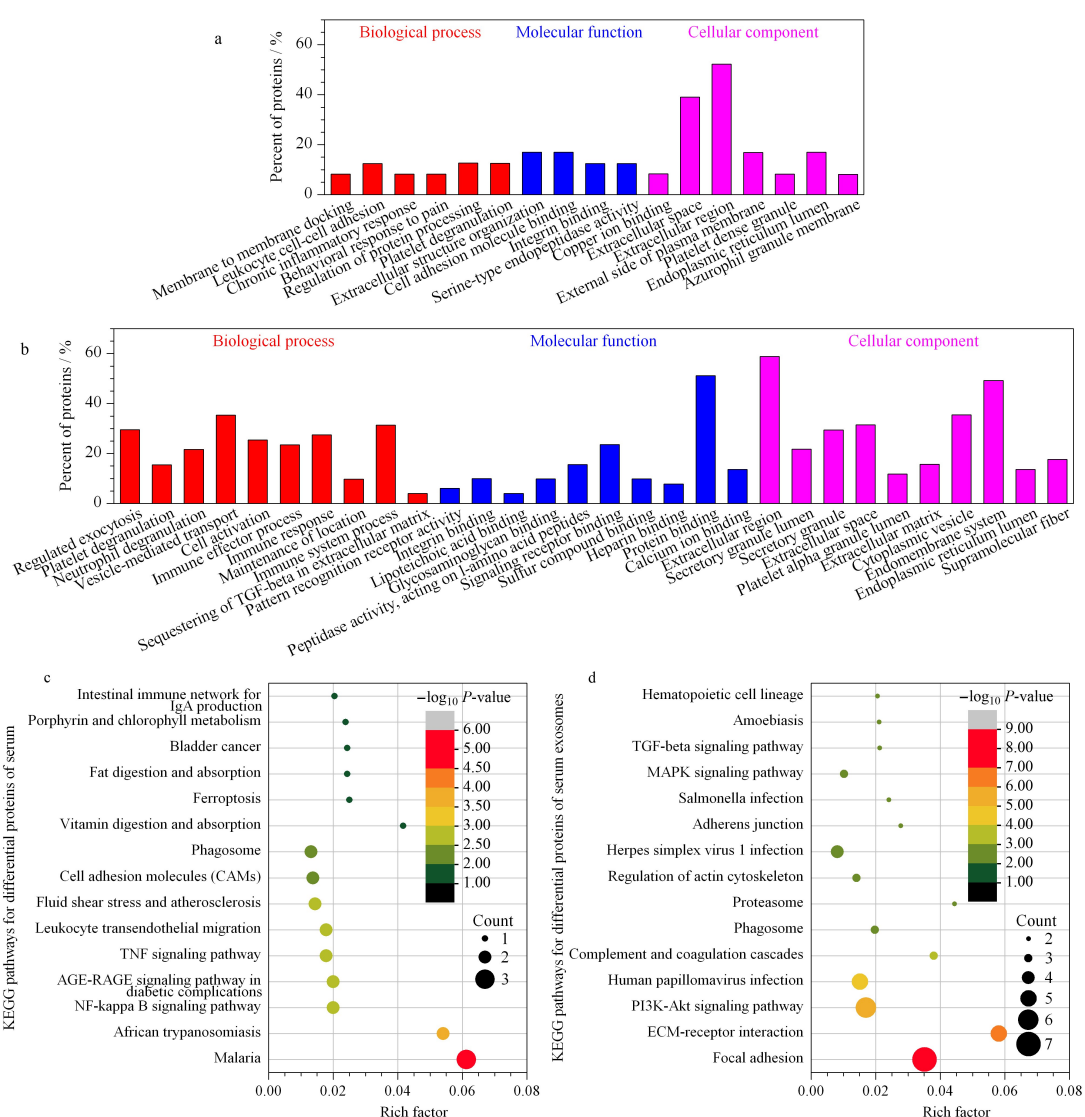
基于血清和血清外泌体样本的HC与iCCA组间差异蛋白质的生物信息分析

在肿瘤微环境中,血小板脱粒过程能增强血小板活化和聚集形成的能力,促使癌栓形成,帮助肿瘤细胞逃脱免疫攻击及其转移行为;同时,血小板释放的颗粒中的促血管生成因子还可促进肿瘤血管的生成,为肿瘤的发生、发展和转移提供生存环境^[33,34]^。

NF-κB信号通路在癌症中具有双重作用,激活NF-κB信号通路一方面可以通过提高细胞毒性免疫细胞对癌细胞的活性以靶向消除转化的细胞等方式发挥免疫防御作用;另一方面则能够在多种类型的癌症中通过控制上皮间质转化或通过血管内皮生长因子及其受体上调控制肿瘤血管形成等过程以促进肿瘤进展^[35]^。如在iCCA中,NF-κB信号通路的活化能够上调CyclinD1等周期促进蛋白活化或抑制Caspase-3和Caspase-8蛋白的活化从而促进肿瘤细胞增殖^[36]^。

中性粒细胞的功能高度依赖于其细胞质颗粒的组成,这些颗粒在中性粒细胞活化后能够释放至细胞外,即中性粒细胞脱粒过程,而颗粒中的许多活性物质已被研究证明与肿瘤的发展有关,如中性粒细胞中预先储存的MMP-9能够通过脱颗粒作用释放至肿瘤微环境中从而高度诱导血管生成,促进肿瘤进展^[37]^。

补体和凝血级联信号通路在免疫反应和凝血过程中起着重要作用。同时,补体系统的激活也会增强肿瘤的恶性生物学行为,如促肿瘤炎症、C3AR介导的肿瘤生长和上皮间质转化、C7和C1Q介导的肿瘤组织沉积以及C1Q介导的血管形成等。此外,肿瘤细胞可以通过SERPINE1和MET癌基因的表达与凝血系统相互作用,并导致SERPINE1显著上调,而SERPINE1则可以通过Fas/Fas-L、Caspase-3和PI_3_K/AKT等途径引起肿瘤的耐药性^[38]^。上述结果表明,在iCCA患者中发现的差异表达蛋白质与iCCA患者的肿瘤发生、发展和转移等过程密切相关,因此差异蛋白质富集到的生物过程和信号通路对探究疾病的发病机制和开发新的治疗方法能够起到借鉴作用。

此外,由图6可知,在研究的两种类型样本中,由于基于血清样本筛选出的差异蛋白质数量较少,因此富集到的生物过程、分子功能和细胞成分的信息也较少(不足10条,已列出获得的所有条目),且信号通路与癌症的相关性也较基于血清外泌体差异蛋白质富集的信号通路与癌症的相关性略低。这是因为外泌体在形成过程中,通过两次质膜内陷以及与内质网、反高尔基网和线粒体等细胞内膜系统产生的早期分选内体融合等过程与细胞进行物质交换,从而携带母细胞信息,并通过胞吐过程释放至胞外。在癌症进展过程中,肿瘤细胞分泌的外泌体可通过其内容物质影响肿瘤形成、生长和转移以及对治疗的抗性等,如乳腺癌细胞源性外泌体内含的miR-105可通过抑制ZO-1表达而损害血管完整性和增强血管通透性导致肿瘤转移^[1]^。因此,相比于无关信息较多的血清样本(当蛋白质量一致时),分析特异性更强的外泌体样本内含的生物活性物质组成(核酸、蛋白质和代谢产物等)可以直观地反映出机体和疾病的不同状态,获取与疾病相关性更嘉的生物学信息,能更有力地解释疾病的发病机制等过程,进一步表明了基于外泌体诊断的特异性和敏感性以及外泌体作为组学分析样本时的优越性,证明了外泌体的应用价值。

## 3 结论

本文通过从血清中分离外泌体并对其进行表征,分别对血清和血清外泌体的蛋白质组进行分析,并将其应用于肝内胆管癌的诊断、潜在标志物发现和探究疾病发生、发展和转移等机制的研究。通过分析获得的无标记定量蛋白质组对健康组和肝内胆管癌患者组进行多维统计分析、差异蛋白质筛选和差异蛋白质生物信息分析,对肝内胆管癌的疾病机制有了进一步的了解。同时,与血清蛋白质组分析相比,血清外泌体蛋白质组分析能够获得更多差异蛋白质和生物学信息;虽然两者获得的信息有所差异,但是也证明了外泌体作为组学分析样本的价值及在诊断领域应用的潜力。
